# On the Construction and Sanitary Arrangements of Barracks, and Hospitals

**Published:** 1855-06

**Authors:** Neil Arnott, B. G. Babington, James Bird, Alexander Browne, W. H. Burrell, John Davy, R. D. Grainger, Waller Lewis, John Liddell, Ranald Martin, J. O. M'William, G. Milroy, E. A. Parkes, T. Southwood Smith, G. Brown, J. Monro, Th. Richardson

**Affiliations:** Sur. h.-p. 37th Reg.; Staff Surgeon, late P. M. O. Eastern Army.; Inspector-General of Hospitals.; Lecturer on Physiology St. Thomas's Hosp.; Metropolitan Commissioner of Sewers.; Royal Hospital, Greenwich.; Govern^t^. Med. Inspector in Jamaica, 1851.; Physician University College Hospital.; Surgeon-Major Grenadier Guards.; Surg.-Major Coldstream Gds.; Surgeon-Major Scots Fusileer Guards.


### ORIGINAL COMMUNICATIONS.

### ON THE CONSTRUCTION AND SANITARY ARRANGEMENTS OF BARRACKS, AND HOSPITALS.

*The Memorial of the undersigned Physicians and Surgeons to the* RIGHT HON. LORD PANMURE, K.T., *Her Majesty's Secretary of State for War,*

Sheweth,—that

I. Your memorialists, in the practice of their profession as medical officers of Her Majesty's army and navy, or of the Hon. East India Company's army, or in official civil service, have long had their attention specially drawn to the investigation of the causes which affect the public health, and tend to produce disease in establishments where large numbers of persons are congregated together.

II. The health of the inmates of such establishments depends mainly upon the observance of certain hygienic conditions which the science of medicine has pointed out, and experience has proved to be necessary for the due performance of the functions of life.

III. The amount of sickness and mortality among the general population of Great Britain is at all times far greater than it ought to be, in consequence principally of the constant prevalence of different epidemic diseases, especially of fevers and bowel complaints, arising from or aggravated by the neglect of sanitary precautions within and around the dwellings of the people.

IV. The healthiness of most of the workhouses and prisons of this country has been, of recent years, very greatly increased by the sanitary improvements which have been introduced into them upon the recommendation of experienced medical officers.

In consequence of these improvements, typhus fever—the existence of which is one of the surest tests of local insalubrity—has been to a great extent banished from among their resident inmates; and the frequency and virulence of bowel disorders and other zymotic diseases have been much diminished.

V. Still more decisive evidence of the efficacy of such measures, in the prevention of disease and in lowering the rate of mortality in similar establishments, has, of late years, been afforded by the experience of the Metropolitan Association for Improving the Dwellings of the Industrious Classes.

During the three years 1850, 51, and 52, the average mortality among the residents—consisting chiefly of the better class of mechanics and artisans, and including a large number of young children, among whom the proportion of deaths is always high—was only 13 and a fraction per 1000; and in 1853, when the whole of the establishments of the Association were in full occupancy, it was much lower, while that of the metropolis generally was at the rate of 22 deaths per 1000.

Not a single case of typhus fever has occurred in any of the improved dwellings since they were first opened.

VI. This remarkable healthiness and exemption from fever have, in the opinion of your memorialists, arisen from the adoption of certain definite sanitary arrangements in the construction of these buildings, viz.:

1. The thorough subsoil drainage of the site.
2. The free admission of air and light to every inhabited room.
3. The abolition of the cesspool and the substitution of the water-closet, with complete house drainage.
4. The abundant supply of pure water.
5. Means for the immediate removal of all solid house refuse, not capable of suspension in water and of being carried off by water.

VII. The average rate of mortality among the troops in the United Kingdom is excessive, being 17 per 1,000 of the whole force, while that among the general civil population at the same period of life—viz., between 20 and 40 years of age—is not quite 12 per 1,000, notwithstanding the abounding preventible causes of ill health among the working classes, especially in large towns.

In the metropolitan police force, which consists of men of about the same age as soldiers, and who are equally exposed to frequent night duty, the rate of mortality during the 10 years 1840-49 did not exceed 7½ per 1,000 among an average force of 4,895 men—or less than one-half the mortality among the troops.

VIII. A large amount of the mortality among the troops of the United Kingdom is caused by continued fever; which, as already stated, has been in a great measure banished even from our work-houses and prisons.

The proportionate mortality from this disease alone in the army at home is double that among the civil population of 24 large towns; and in one of the metropolitan barracks it has, on the average of 10 years, 1837-46, been as high as at the rate of 4 deaths per 1,000 of the strength—an enormous proportion, four or five times as great as that among the general population of England.

IX. The rate of mortality among the troops in the United Kingdom from diseases of the stomach and bowels, including diarrhœa and dysentery, is much higher than among the population of our towns at the same periods of life.

X. The troops in Great Britain have on several occasions suffered severely from Asiatic cholera, the fatal effects of which disease universal experience has shewn to be so intimately connected with the sanitary condition of dwellings.

During the visitation of 1849, from the beginning of July to the end of the epidemic, in a force of 4,469 men in the different barracks of the metropolis, there occurred 69 cases and 27 deaths; while not more than one case of cholera occurred among the inmates, 795 in number, of the metropolitan buildings or modellodging houses, although several of these buildings are situated in neighbourhoods where the pestilence raged with severity, and many of the residents, being artisans, were much exposed in their daily occupations to unfavourable influences.

Moreover, in 7 of the metropolitan prisons, having between 2,000 and 3,000 inmates, there were not more than 3 cases and 2 deaths during the whole epidemic. This fact is the more worthy of notice, as some of these prisons had suffered severely in the visitation of 1832, since which time important improvements had been effected in their sanitary condition at the instance of the medical officers.

The complete exemption, too, of Bethlem Hospital, and of the Hanwell Asylum, containing together 1,361 patients besides attendants, is a striking evidence of the same nature.

XI. Besides these diseases already mentioned, other maladies, which at all times cause great mortality and a large amount of invaliding among the troops in the United Kingdom, as disorders of the respiratory organs, are much aggravated, if not directly induced, by local causes affecting the health of the soldier; and among these causes, none is so detrimental as the mal-sanitary condition of many of the existing barracks, guard-houses, &c.

XII. The different proportion of sickness and mortality in the different metropolitan barracks affords the strongest presumption that this difference arises from the operation of injurious local causes in connection with these barracks upon the health of the men.

XIII. A similar faulty state of things, as respects the barrack and other accommodation, exists in almost every colonial possession of the British crown, to the serious injury of the health, the sacrifice of much life, and the consequent impairment of the effective strength of the troops, as well as involving a very heavy expenditure to replace them.

XIV. Should your Lordship desire to be provided with specific proofs of the above allegations respecting the excessive mortality in the army both at home and abroad, your memorialists are prepared to furnish detailed information on the subject.

XV. Your memorialists are convinced that the required improvements in the barrack &c. accommodation, and other physical comforts of the troops, would not only much diminish the existing amount of sickness, invaliding and death, and thus eventually lead to a great saving of national expenditure, but would also react most advantageously on the moral and intellectual character of the soldier, and on the general efficiency and welfare of the service.

The besetting vice of drunkenness would doubtless be much diminished, from the increased salubrity of the air of his dwelling, and the consequent absence of that depression which invariably results from residence in a vitiated atmosphere.

XVI. As it has been announced in Parliament that several large barracks are about to be erected in different parts of the country, your memorialists would respectfully urge upon the Government that it is highly desirable that all the late improvements in the construction of buildings bearing upon health should be introduced, more especially as these improvements are in themselves inexpensive; and they would submit that as regards the site, preparation of the ground, and the structural arrangements and appliances of barracks, guard-houses, military hospitals, and prisons, medical men, who have paid special attention to the subject, should be consulted, in order that in a matter of so much public interest the army should have the advantage of all the experience derived from the progress of sanitary science.

(Signed) NEIL ARNOTT, M.D., F.R.S.
B. G. BABINGTON, M.D., F.R.S.,
JAMES BIRD, M.D., F.R.C.S., &c.
ALEXANDER BROWNE, M.D., *Sur. h.-p. 37th Reg.*
W. H. BURRELL, M.D.,
*Staff Surgeon, late P. M. O. Eastern Army.*
JOHN DAVY, M.D., F.R.S. L. & Ed.,
*Inspector-General of Hospitals.*
R. D. GRAINGER, F.R.S.,
*Lecturer on Physiology St. Thomas's Hosp.*
WALLER LEWIS, M.B., F.G.S.,
*Metropolitan Commissioner of Sewers.*
JOHN LIDDELL, C.B., M.D., F.R.S.,
*Royal Hospital, Greenwich.*
RANALD MARTIN, F.R.S.
J. O. M'WILLIAM, M.D., F.R.S.
G. MILROY, M.D.,
*Govern^t. Med. Inspector in Jamaica,* 1851.
E. A. PARKES, M.D.,
*Physician University College Hospital.*
T. SOUTHWOOD SMITH, M.D.
G. BROWN, *Surgeon-Major Grenadier Guards.*
J. MONRO, M.D., *Surg.-Major Coldstream Gds.*
TH. RICHARDSON,
*April* 11, 1855. *Surgeon-Major Scots Fusileer Guards.**

---

* On the 23rd of May, Drs. Arnott, Brown, Monro, Bird, Lewis, M'William, and Milroy, had the honour of an interview with Lord Panmure upon the subject of the above memorial.

At this interview his lordship expressed his high sense of the importance of the subject, and of the value of the information and suggestions brought under his notice.

The memorial had been referred by him to Dr. Graham Balfour, of the Royal Military Asylum, for consideration, and that gentleman had on nearly all the material points confirmed the statements contained in it.

Lord Panmure said that he entirely coincided in the recommendation that no barrack or military hospital should ever be built without the plans as to the site, construction, and sanitary arrangements having been previously submitted to a medical opinion. The memorial would be transmitted to the Barrack Committee now sitting, and the attention of the committee drawn to the offer made by the memorialists to afford detailed information respecting the condition of existing barracks and hospitals at home and in the colonies, if such information was desired.

